# Political decision-makers and mathematical modellers of infectious disease outbreaks: the sweet spot for engagement

**DOI:** 10.1136/bmjgh-2024-015155

**Published:** 2024-09-24

**Authors:** Sabine L van Elsland, Paula Christen

**Affiliations:** 1 MRC Centre for Global Infectious Disease Analysis, School of Public Health, Imperial College London, London, UK; 2 Center for Epidemiological Modelling and Analysis (CEMA), University of Nairobi, Nairobi, Kenya

**Keywords:** COVID-19, Decision making, Global health, Health policy, Epidemiology

**Summary box**

- Knowledge translation from science to policy is well-studied in clinical and general public health contexts. There is no consensus on best practices specific to advanced analytics to inform decisions during public health emergencies and the processes for their translation into policy are not well-established or institutionalised.
- During public health emergencies, with the prime example of the COVID-19 pandemic, scientists have had a shared vested interest in the political decisions that were made based on the evidence they produced.
- This context presents a unique case study to learn about knowledge translation from advanced analytics in public health emergency decision-making.
- We highlight the need for data harmonisation, capacity building and improved communication to strengthen the translation of scientific evidence into action.
- We emphasise the need for systematic research on knowledge translation to be conducted while institutional memory and relationships between scientists and policymakers are still fresh. Knowledge translation processes that were effective during the pandemic should be systematically identified and institutionalised for future preparedness.
- The commentary presents research priorities for practice (stressing the necessity to institutionalise capacity for advanced analytics and ensure the knowledge translation process and the need for the development of guidelines), and policy (proactive investments and joint efforts are required to improve knowledge translation mechanisms and enable better informed and timely decision-making in future public health emergencies).

The time to systematically learn from the COVID-19 pandemic is now, while COVID-19 is no longer considered a public health emergency, yet with the risk of another pandemic in the near future. The wide variety of response strategies to the COVID-19 pandemic has resulted in a natural experiment of how scientific evidence influences both public health and policy decisions as well as the course of a global crisis. Scientific evidence in the form of advanced analytics received attention outside the academic community like never before and played a pivotal role in political decision-making processes.

The concept of evidence-informed decision-making is not new; think about evidence-based medicine dating back to the 1980s where, for example, randomised clinical trials, systematic reviews and meta-analyses became the ‘gold standard’ for patient-level decision-making.^1^ In the policy context, knowledge translation or evidence-informed decision-making can be defined as the synthesis, exchange and application of knowledge by relevant stakeholders to accelerate the benefits of global and local innovation in strengthening health systems and improving people’s health.^2^ This occurs within a complex system of interactions among researchers and users.^3^ Yet, our understanding of the role of advanced analytics and mathematical models as the basis for evidence-informed decision-making is still in its infancy.

The WHO Hub for Pandemic and Epidemic Preparedness together with the Medical Research Council Centre for Global Infectious Disease Analysis at Imperial College London organised a workshop in 2023 to better understand the context and ways in which advanced analytics were used for decision-making during the COVID-19 pandemic. During this workshop, clear opportunities to strengthen data-to-decision pathways were identified.^4^

Structural changes cannot be made during an emergency response; proactive investments are needed to institutionalise processes and systems that enable effective knowledge translation. Here, we discuss three aspects of particular relevance for knowledge translation of advanced analytics for policy in preparation for future public health emergencies: data harmonisation, capacity and communication.

First, during public health emergencies or outbreaks, data collection, ethics and General Data Protection Regulations need to be harmonised. Relevant considerations are what data needs to be collected at what time, by whom, at what aggregation and how this information is linked. For example, Singapore had an accelerated digitalisation and datafication of contact tracing^5^ while Germany had a more fragmented data architecture inhibiting effective linkage for epidemiological investigation.^6^

Second, global collaborations across scientific groups were unparalleled. However, modelling capacity overall was limited and unevenly distributed geographically, concentrated among Western European and Northern American-based individuals or academic research institutions. Sustainable knowledge translational processes within countries need to be institutionalised. Global and regional guidelines, which can be adapted to local contexts, need to be drawn up including a good understanding of what policy-relevant advanced analytics are.

Lastly, while there was unprecedented interaction between scientists and policymakers,^7^ the exact processes underpinning both the formal and informal communication routes differed between countries and were difficult to unravel. A Cochrane review is currently identifying approaches for knowledge translation from advanced analytics to policy.^8^ In order to leverage the valuable relationships that were established during the pandemic for future preparedness, it is important to shed light on the ‘black box’ that sits between the scientists, decision-makers and intermediaries. We need to understand what is shared, by whom, how and how often? What were the most effective modes of communication? What questions tackled by scientists were policy relevant? What questions posed by policymakers can and cannot be answered by advanced analytics? What data were required to answer these questions?

Freedom of speech is another communication challenge that needs to be addressed. While scientists in some countries communicated only with decision-makers behind closed doors, others became public figures and shared scientific findings with a wide audience in media and through other platforms. The extent to which these different approaches impacted the public and policy decision-maker’s understanding of the evidence, their behaviour and decisions made, is unclear. Science was often not made available nor communicated transparently to the public, with policy decision-makers gatekeeping evidence. Scientists in some countries were simply not permitted to communicate the extent of uncertainties or explain knowledge gaps.^4^

While the pandemic institutional memory and relationships between scientists and policymakers are fresh, we need to systematically identify knowledge translation processes that worked and improved public health. The window to constructively take learnings from this experience and take action to build on individual expertise gained during this time is closing. Joint scientific and policy efforts and funding are required to improve and institutionalise knowledge translation processes.

## Data Availability

There are no data in this work.
